# Melodies as Maximally Disordered Systems under Macroscopic Constraints with Musical Meaning

**DOI:** 10.3390/e21050532

**Published:** 2019-05-25

**Authors:** Jorge Useche, Rafael Hurtado

**Affiliations:** Departamento de Física, Universidad Nacional de Colombia, Carrera 45 No. 26-85, Bogotá 111321, Colombia

**Keywords:** consonance, Kullback–Leibler divergence, melody, musical interval, relative entropy

## Abstract

One of the most relevant features of musical pieces is the selection and utilization of musical elements by composers. For connecting the musical properties of a melodic line as a whole with those of its constituent elements, we propose a representation for musical intervals based on physical quantities and a statistical model based on the minimization of relative entropy. The representation contains information about the size, location in the register, and level of tonal consonance of musical intervals. The statistical model involves expected values of relevant physical quantities that can be adopted as macroscopic constraints with musical meaning. We studied the occurrences of musical intervals in 20 melodic lines from seven masterpieces of Western tonal music. We found that all melodic lines are strictly ordered in terms of the physical quantities of the representation and that the formalism is suitable for approximately reproducing the final selection of musical intervals made by the composers, as well as for describing musical features as the asymmetry in the use of ascending and descending intervals, transposition processes, and the mean dissonance of a melodic line.

## 1. Introduction

Many quantitative analyses in music have been carried out using different elements as building blocks, or “units of context”, which allow the message of a musical piece to be apprehensible at different time scales [1]. Common choices for these “units of context” are single pitches (ignoring or taking into account the *chroma* properties [2,3]), single musical notes (i.e., pitch and rhythm values), pairs of pitches or musical intervals (either harmonic or melodic), triplets of pitches between contiguous notes, and chords [1,2,3,4,5,6,7,8,9]. In the case of musical intervals (from now on referred to as intervals), quantitative analyses frequently employ parameters that describe their psychoacoustic properties, such as the sizes of intervals (commonly measured in tones or semitones), the ratio of the fundamental frequencies of both pitches (commonly measured in units of cents), and the difference between the fundamental frequencies [10,11].

Analyses based on statistical methods can capture information about musical features, such as the style of a musical piece, the composer, and even the emotions conveyed [1,3,4,5,6,7,8,9,12,13,14,15,16,17,18,19,20]. Several statistical analyses employ successive pitches as units of context. George Kingsley Zipf studied the frequency of occurrences of melodic intervals in masterpieces of Western music, and he reported that the frequency of occurrences of ascending and descending intervals is almost inversely proportional to their size [4]. Vos and Trost studied music from 13 great composers of Western academic music, the Beatles, and folk music, finding that the proportion between musical interval sizes is too complex to be represented by a simple exponential or power law function. They also reported an asymmetry in the use of ascending and descending intervals [5]. Gunnar Niklasson and Maria Niklasson studied the occurrences of melodic intervals as a function of their size, finding long-tailed Levy-stable distributions that they associated to a “music walk” between successive pitches, in analogy with a random walk [19]. In the framework of a network analysis, Liu, Small, and Tse studied the connectivity properties of complex networks representing the successive notes of musical pieces, finding scale-free behavior in the nodal degree for several sets of academic and popular music [8].

From the information theory perspective, there are many works devoted to analyzing, classifying, and generating music [21,22,23,24,25,26,27,28,29]. A seminal work by Cohen establishes the basic concepts for applying information theory to music [30]. In this approach, entropy has been used to measure the amount of information conveyed to a listener in a sequence of events organized in time [27]. Many works have used this concept in music, for example: Pinkerton wondered how entropy must be measured in melodies and how large it should be [21]; Youngblood [22], and Knopoff and Hutchinson [25] used entropy in order to identify musical styles. Manzara, Witten, and James used entropy to characterize the short- and long-term structures in chorales of J. S. Bach. [27]. Dubnov, Assayag, and El-Yaniv used entropy to characterize sequences of pitches in terms of their statistical source coding, and to generate aleatoric melodies [26]. Güngör Gündüz and Ufuk Gündüz studied the evolution of the entropy associated to the transitions between pitches during the progress of a melody, and they found that it increases up to a limiting value, which is smaller than the entropy of a random melody [28]. Cohen posted various criticisms about the application of information theory in music [30]. For example: Markov sources cannot generate some sequences found in music, the assumption concerning that a probability distribution corresponds to the listener’s expectations is difficult to hold as the expectations change dynamically through the musical piece, the ergodicity of the source should be considered only as an approximation, the impossibility of an infinite memory capacity in the listener must be taken into account, and finally, the assumption concerning the stationarity of the source is difficult to prove [30].

With respect to the melodic interval size, David Huron carried out a study of nearly 10,000 Western musical themes, finding that the average melodic interval size is slightly smaller in pieces written in a minor mode than in those written in a major mode. This result was interpreted by the author as a relation between sadness and small values for the average melodic interval size [6]. Huron also found that themes in a minor mode have slightly lower pitches on average in comparison with major ones [14], which suggests that the sizes of intervals and their locations in the register are important for conveying musical information.

The concept of interval size captures relevant information on musical features. However, it misses information concerning the locations of intervals in the register and, hence, on the level of tonal consonance [11] and musical processes, such as the transposition [31]. Here, we propose a representation of intervals that contains information on their sizes and locations in the register. This representation utilizes the fundamental frequencies of pitches and relates the tonal consonance properties of intervals to the work carried out by the composer choosing the intervals for a piece. This formalism is employed to study melody through the analysis of 20 melodic lines from seven masterpieces of Western tonal music, including the development of a theoretical model based on the relative entropy extremalization that reproduces the main features observed in real melodic lines.

This paper is organized as follows. Section 2 presents the microscopic representation of intervals and describes how to construct their macroscopic observables. Section 3 describes how to measure levels of tonal consonance using the representation proposed. Section 4 introduces macroscopic observables in melody. Section 5 and Section 6 present an application to real melodic lines and a statistical model that reproduces the main experimental findings. The final section presents the conclusions.

## 2. Microscopic Representation and Macroscopic Observables of Intervals

This section presents the microscopic representation of musical intervals using physical quantities, the expected values of the relevant quantities, the mathematical description of transposition processes in this representation, and an analysis of distinguishability of intervals.

### 2.1. Interval Size and Its Relation to the Fundamental Frequency of Pitches

Many musical systems employ discrete sets of sounds produced by musical instruments, which are usually grouped into musical scales. Frequently, these sets of sounds are selected in such a way as to yield a large number of consonant combinations when two or more elements are produced together [10]. Pythagoras posted the first known mathematical rule to produce musical scales when he found that two sounds emitted simultaneously by vibrating strings of equal tension and density produce a pleasant sensation when the ratio between their lengths ($l_{i}/l_{j}$) and, hence, between their fundamental frequencies ($f_{j}/f_{i}$) corresponds to the ratio between two small natural numbers $l_{i}/l_{j}=f_{j}/f_{i}=n/m$ [10,32,33]. The frequency ratio is the first known parameterization of consonance in terms of physical quantities. Two well-known scales based on the Pythagoras rule are the just and the Pythagorean [10,32].

Ordering the *R* pitches produced by a musical instrument tuned to a particular musical scale from the lowest to the highest fundamental frequency leads to a collection of pitches $\left\{f_{1},f_{2},\ldots ,f_{i},\ldots ,f_{R}\right\}$ with $f_{1}<f_{2}<,\ldots <f_{i}<\ldots <f_{R}$. The interval size *L* associated to a pair of pitches $f_{i}$ and $f_{j}$ is defined for many musical scales as $L\equiv L(f_{i},f_{j})=j-i$. The magnitude of *L* determines the plain distance between pitches, and its sign is meaningful for successive pitches, distinguishing the chronological order of their appearances. Intervals with the same size *L* can be produced in different locations of the register. In analogy with the concept of degeneracy used in physics, this quantity can be considered as degenerated with a value that is equal to the total number of such intervals. For complex tones, such as the sounds produced by musical instruments that can be described as a superposition of several pure tones, Plomp and Levelt found that an interval with a given frequency ratio $f_{j}/f_{i}$ might be more or less consonant depending on its location in the register [11]. In many musical cases, there is a one-to-one correspondence between *L* and $f_{j}/f_{i}$, for example, in an equal-tempered system.

**Figure 1:**
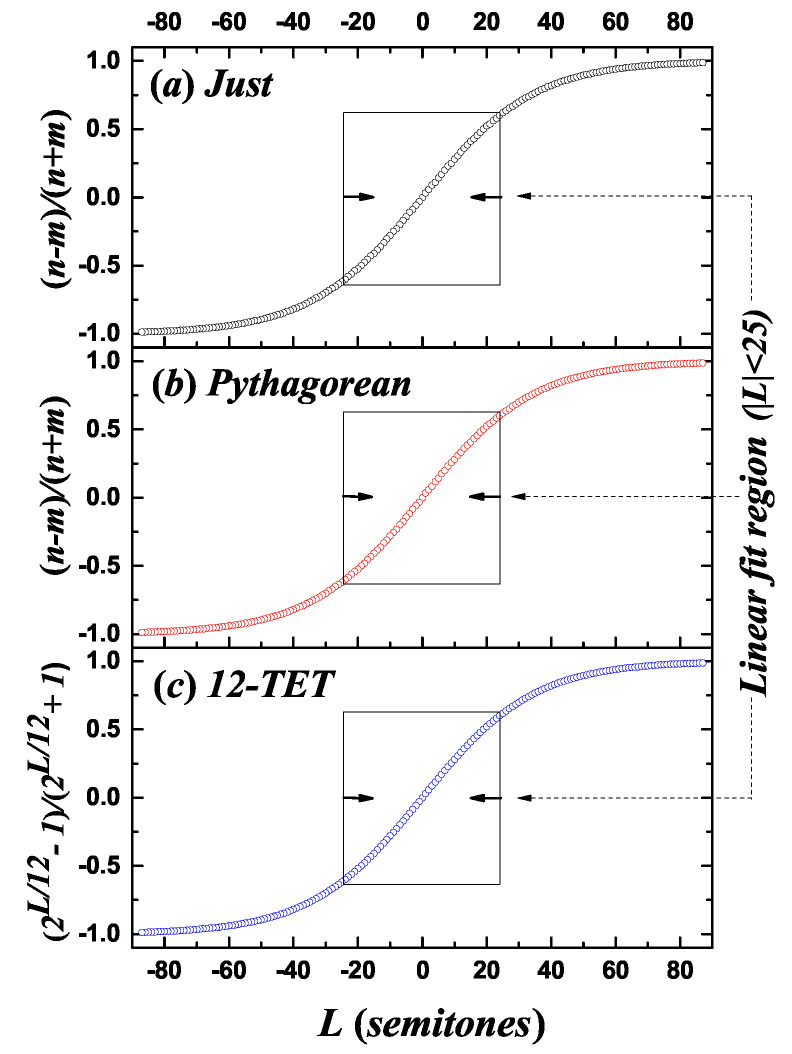
Relation between musical scale parameters and the interval size for the (**a**) just, (**b**) Pythagorean, and (**c**) 12-TET scales, with an interval size from –87 to 87 semitones (representing a typical piano). The linear fit corresponds to interval sizes between –24 and 24 semitones.

The Pythagoras rule can be expressed as the frequency difference:

(1) $$f_{j}-f_{i}=\left[\left(n-m\right)/\left(n+m\right)\right]\left(f_{j}+f_{i}\right),$$

where for the just and Pythagorean scales, the quantity (n−m)/(n+m) depends on the size of the interval *L* (see Figure 1).

The 12-tone equal-tempered (12-TET) scale belongs to the equal tempered system and has been widely utilized in Western tonal music. This system is based on a different mathematical rule, $f_{i}=f_{1}\sqrt[{h}]{2^{i}}$, where *h* is a natural number (h=12 for the 12-TET) and $f_{1}$ is a reference frequency. In this system, the frequency ratio is given by:

(2) $$f_{j}/f_{i}=\sqrt[{h}]{2^{j-i}}=\sqrt[{h}]{2^{L}},$$

and an equivalent expression to (1) is:

(3) $$f_{j}-f_{i}=\frac{2^{L/h}-1}{2^{L/h}+1}\left(f_{j}+f_{i}\right).$$

Equation (3) approximately holds for the just and Pythagorean scales, taking $\left(n-m\right)/\left(n+m\right)=\left(2^{L/b}-1\right)/\left(2^{L/b}+1\right)$ and using the most common values of *n* and *m* related to each interval size *L* in the just and Pythagorean scales (see Supplementary Table S1) [32], then for a register with 88 pitches, the obtained fit parameters are as follows:

- In the just scale, $b=12.0040\pm 6.8\times 10^{-3}$ with a determination coefficient $R^{2}\approx 1$;
- In the Pythagorean scale, $b=11.9767\pm 4.9\times 10^{-3}$ with $R^{2}\approx 1$;
- In the 12-TET, b=12.

The expression $\left(2^{L/b}-1\right)/\left(2^{L/b}+1\right)$ can be written as a linear function of *L* in a broad region, see Figure 1. The second-order term of the Taylor expansion around L=0 vanishes, and the first-order term leads to $\left(2^{L/b}-1\right)/\left(2^{L/b}+1\right)\approx cL$, with c=(ln2)/(2b).

In many musical cases, the sizes of intervals are smaller than or equal to two octaves, such as in the case of melodic intervals in typical melodic lines [31]. For the case that −24≤L≤24, the fit parameters are given as follows:

- For the just scale, $c=2.632\times 10^{-2}\pm 1.52\times 10^{-4}$ with a determination coefficient $R^{2}=0.998$;
- For the Pythagorean scale, $c=2.642\times 10^{-2}\pm 1.55\times 10^{-4}$ with $R^{2}=0.998$;
- For the 12-TET scale, $c=2.635\times 10^{-2}\pm 1.48\times 10^{-4}$ with $R^{2}=0.998$.

With these results, Equations (1) and (3) can be expressed as:

(4) $$f_{j}-f_{i}\approx cL\left(f_{j}+f_{i}\right)=2cLX,$$

where $X=(f_{j}+f_{i})/2$ is the center frequency, which provides information about the location of an interval in the register [10]. Then, $f_{j}-f_{i}$ is proportional to the product of the interval size *L* and its corresponding location in the register *X*, lifting the degeneration associated to the fact that intervals of the same size might be produced in different locations of the register.

### 2.2. Expected Values with Musical Meaning

Let us suppose that in a musical piece, the probability associated to the frequency of occurrence of each interval of size *L* is known to be $\left\{p_{{}_{L}}\right\}$ with $\sum_{L}p_{{}_{L}}=1$. If the probability $p_{{}_{L}}$ is related to simultaneous pitches, then *L* can be defined as $\left|L|\equiv |L(f_{i},f_{j})|=|j-i\right|$.

Probability distributions (PD) allow us to obtain macroscopic quantities related to specific properties of musical pieces. For example, the average magnitude of the interval size is given by:

(5) $$\langle |L|\rangle =\sum_{L=L_{min}}^{L_{max}}\left|L\right|p_{{}_{L}}.$$

Frequently, different musical instruments have different registers. However, Equation (5) does not capture this information, for example, in a transposition process that moves a set of intervals from one part of the register to another. The expected value of the frequency difference captures information about the locations of intervals in the register:

(6) $$\begin{array}{c} \begin{array}{c} \langle |f_{j}-f_{i}|\rangle =\frac{|f_{j_{1}}-f_{i_{1}}\left|+\right|f_{j_{2}}-f_{i_{2}}\left|+\ldots +\right|f_{j_{N}}-f_{i_{N}}|}{N}=\frac{\sum_{i^{\prime },j^{\prime }}|f_{j^{\prime }}-f_{i^{\prime }}|{|}_{{}_{L_{min}}}+\ldots +\sum_{i^{\prime \prime },j^{\prime \prime }}\left|f_{j^{\prime \prime }}-f_{i^{\prime \prime }}\right|{|}_{{}_{L_{max}}}}{N}\\ =\frac{N_{{}_{L_{min}}}\langle |f_{j^{\prime }}-f_{i^{\prime }}{|\rangle }_{{}_{L_{min}}}}{N}+\ldots +\frac{N_{{}_{L_{min}}}\langle |f_{j^{\prime \prime }}-f_{i^{\prime \prime }}{|\rangle }_{{}_{L_{max}}}}{N}, \end{array} \end{array}$$

where *N* is the total number of intervals, $\sum_{i^{\prime },j^{\prime }}|f_{j^{\prime }}-f_{i^{\prime }}|{|}_{{}_{L_{min}}}+\ldots +\sum_{i^{\prime \prime },j^{\prime \prime }}\left|f_{j^{\prime \prime }}-f_{i^{\prime \prime }}\right|{|}_{{}_{L_{max}}}$ is the sum of the frequency differences of intervals grouped by their size, and $N_{{}_{L_{min}}},\ldots ,N_{{}_{L_{max}}}$ are the total numbers of intervals of each size *L*. Taking $p_{{}_{L}}=N_{{}_{L}}/N$ as the probability of finding an interval of size *L*, the expected value is:

(7) $$\begin{array}{c} \begin{array}{c} \langle |f_{j}-f_{i}|\rangle =\sum_{L=L_{min}}^{L_{max}}\langle |f_{j^{\prime }}-f_{i^{\prime }}{|\rangle }_{{}_{L}}p_{{}_{L}}\;, \end{array} \end{array}$$

where $\langle |f_{j^{\prime }}-f_{i^{\prime }}{|\rangle }_{{}_{L}}$ is the mean value of the frequency differences for a set of intervals of size *L*. The linear approximation leads to:

(8) $$\begin{array}{c} \begin{array}{c} \langle |f_{j}-f_{i}|\rangle \approx 2c\sum_{L=L_{min}}^{L_{max}}{|L|\langle X\rangle }_{{}_{L}}p_{{}_{L}}=2c\sum_{L=L_{min}}^{L_{max}}\left|L\right|p_{{}_{L}}\;, \end{array} \end{array}$$

where $L=L{\langle X\rangle }_{{}_{L}}$ is an effective size containing information about the contribution of the average location in the register. Equation (8) can be considered as an extension of Equation (5) when the average position in the register of each type of interval size ${\langle X\rangle }_{{}_{L}}$ is taken into account. Notice that if all intervals have the same average position in the register $X_{{}_{C}}$, then the expected value $\left|\langle f_{j}-f_{i}\rangle \right|$ is proportional to the expected value 〈|L|〉, being given by $\langle |f_{j}-f_{i}|\rangle \approx 2cX_{{}_{C}}\langle |L|\rangle$. Equation (8) shows that the expected value associated to the frequency differences takes into account the mean location in the register of intervals. However, the diversity of locations in the register for the same interval does not contribute to the expression (8). A quantity that takes into account this diversity can be constructed from Equation (4) as:

(9) $$f_{j}^{2}-f_{i}^{2}=\left(f_{j}-f_{i}\right)\left(f_{j}+f_{i}\right)\approx 4cL{\left(\frac{f_{j}+f_{i}}{2}\right)}^{2}=4cLX^{2}.$$

From the physics perspective, this quantity is proportional to the difference in the average energy densities $\epsilon_{j}-\epsilon_{i}$ for two harmonic waves with equal amplitudes *T* propagating in a medium with density ρ [34]:

(10) $$\epsilon_{j}-\epsilon_{i}=2\pi^{2}\rho T\left(f_{j}^{2}-f_{i}^{2}\right)\;.$$

The expected value of the quantity $f_{j}^{2}-f_{i}^{2}$ in Equation (9) can be written as:

(11) $$\begin{array}{cc} \langle |f_{j}^{2}-f_{i}^{2}|\rangle =\sum_{L=L_{min}}^{L_{max}}\langle |f_{j^{\prime }}^{2}-f_{i^{\prime }}^{2}{|\rangle }_{{}_{L}}p_{{}_{L}} & \approx 4c\sum_{L=L_{min}}^{L_{max}}\left|L\right|{\langle X^{2}\rangle }_{{}_{L}}p_{{}_{L}}=4c\sum_{L=L_{min}}^{L_{max}}\left|L\right|\left({\langle X\rangle }_{{}_{L}}^{2}+\sigma_{{}_{L}}^{2}\right)p_{{}_{L}}\\ & =4c\sum_{L=L_{min}}^{L_{max}}\left|L\right|p_{{}_{L}}\;, \end{array}$$

where the term ${\sigma_{{}_{L}}}^{2}$ represents the dispersion of the intervals of size *L* in the register (measured as a variance) with respect to the average position ${\langle X\rangle }_{{}_{L}}$, and $L=L\left({\langle X\rangle }_{{}_{L}}^{2}+\sigma_{{}_{L}}^{2}\right)$ is an effective size that takes into account the contribution of the average location of intervals in the register as well as their dispersion. Equation (11) can be considered as an extension of Equation (8), when the contribution from the dispersion in the locations of the intervals is taken into account. In the case of just one possible location in the register for each kind of interval of size *L*, ${\sigma_{{}_{L}}}^{2}=0$. In addition, if the average positions in the register for intervals of different sizes are close to each other and they are located around the position $X_{{}_{C}}$, then the first-order term of the Taylor expansion around $X_{{}_{C}}$ leads to ${\langle X\rangle }_{{}_{L}}^{2}\approx 2X_{{}_{C}}{\langle X\rangle }_{{}_{L}}-X_{{}_{C}}^{2}\approx X_{{}_{C}}{\langle X\rangle }_{{}_{L}}$. Hence, these approximations lead to $\langle |f_{j}^{2}-f_{i}^{2}|\rangle \approx 2X_{{}_{C}}\langle |f_{j}-f_{i}|\rangle$.

### 2.3. Transposition Process

In a transposition process, the set of probabilities $\left\{p_{{}_{L}}\right\}$ remains unvaried when the location of the intervals in the register is moved from the original one ${\langle X\rangle }_{{}_{L}}^{O}$ to a new one ${\langle X\rangle }_{{}_{L}}^{N}$. These locations are related as:

(12) $${\langle X\rangle }_{{}_{L}}^{N}=w{\langle X\rangle }_{{}_{L}}^{O}\;;\;w=f_{N}/f_{O}\;,$$

where $f_{O}$ refers to any fundamental frequency in the original location, $f_{N}$ is the corresponding frequency in the new location, and *w* is the interval of the transposition. While the observable 〈|L|〉 remains unchanged after the transposition process, $\langle |f_{j}-f_{i}|\rangle$ changes as follows:

(13) $$\langle |f_{j}-f_{i}{|\rangle }_{N}=w\langle |f_{j^{\prime }}-f_{i^{\prime }}{|\rangle }_{O}\;,$$

where $\langle |f_{j^{\prime }}-f_{i^{\prime }}{|\rangle }_{O}$ and $\langle |f_{j}-f_{i}{|\rangle }_{N}$ denote to the expected values in the original and new locations of the register, respectively.

In the case of an observable $\langle |f_{j}^{2}-f_{i}^{2}|\rangle$, the variance in the new location ${\left(\sigma_{{}_{L}}^{2}\right)}_{N}$ changes with respect to the variance in the original location ${\left(\sigma_{{}_{L}}^{2}\right)}_{O}$ by the square of the interval of the corresponding transposition $w^{2}$:

(14) $${\left(\sigma_{{}_{L}}^{2}\right)}_{N}=w^{2}\left[{\left(\sigma_{{}_{L}}^{2}\right)}_{O}\right].$$

Because ${\langle X\rangle }_{{}_{L}}^{2}$ also scales with $w^{2}$, in a transposition process, the ratio ${\langle X\rangle }_{{}_{L}}^{2}/\sigma_{{}_{L}}^{2}$ remains unchanged, and the expected value $\langle |f_{j}^{2}-f_{i}^{2}|\rangle$ scales as:

(15) $$\langle |f_{j}^{2}-f_{i}^{2}{|\rangle }_{N}=w^{2}\langle |f_{j^{\prime }}^{2}-f_{i^{\prime }}^{2}{|\rangle }_{O}\;,$$

where $\langle |f_{j^{\prime }}^{2}-f_{i^{\prime }}^{2}{|\rangle }_{O}$ and $\langle |f_{j}^{2}-f_{i}^{2}{|\rangle }_{N}$ are the expected values in the original and new locations, respectively.

### 2.4. Distinguishability of Pairs of Pitches

So far, it has been shown that the quantities $f_{j}-f_{i}$ and $f_{j}^{2}-f_{i}^{2}$ distinguish between intervals of the same size in different locations in the register (Equations (4) and (9)).

**Figure 2:**
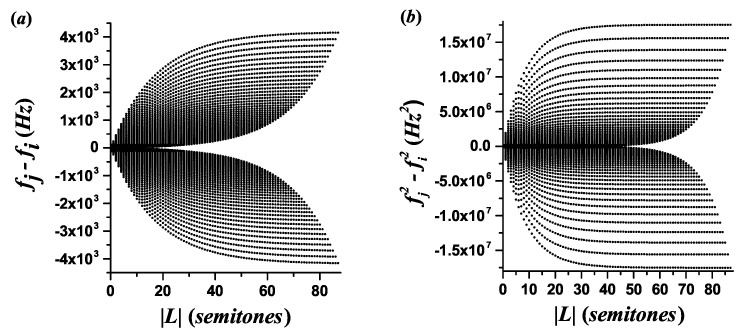
Relation between the quantities $f_{j}-f_{i}$ and $f_{j}^{2}-f_{i}^{2}$ and the magnitude of the interval size |L| in semitones for $f_{j}>f_{i}$, shown in panels (**a**,**b**), respectively. The register corresponds to a typical 88 key piano. The upper branch comes from j=88 (highest pitch), and *i* varies from 88 to 1. The tuning comes from the frequency relation for the 12-TET scale with A=440 Hz.

Figure 2 illustrates the dependence of $f_{j}-f_{i}$ and $f_{j}^{2}-f_{i}^{2}$ on the magnitude of the interval size |L| for the 12-TET scale tuned with A=440 Hz. Considering the orders of magnitude of the values and the relative separations between branches, this figure indicates that the quantity $f_{j}^{2}-f_{i}^{2}$ has a better resolution than $f_{j}-f_{i}$ for distinguishing intervals of equal size in different locations of the register.

The distinguishability of intervals of different sizes in different locations of the register is not evident. The general problem can be formulated independently of the musical scale and the particular tuning as follows: If two pairs of different pitches $\left\{f_{i},f_{j}\right\}$ and $\left\{f_{r},f_{s}\right\}$ produce the same frequency difference or the same difference in the squares of the frequencies, then:

(16) $$f_{j}-f_{i}=f_{s}-f_{r}\;;\;f_{j}^{2}-f_{i}^{2}=f_{s}^{2}-f_{r}^{2}\;;\;\mathrm{for}\;f_{j}>f_{i}\;\left(i.e.,\;j>i\right)\;\mathrm{and}\;f_{s}>f_{r}\;\left(i.e.,\;s>r\right).$$

When $f_{j}<f_{i}$ and $f_{s}<f_{r}$, Equation (16) can be transformed into a positive equality by changing the index order i↔j;s↔r. Then, both cases are equivalent.

The solution of these equations can be formulated in terms of the frequency ratio of the fundamental frequencies, instead of the specific values of the frequencies. In many cases, as for example in the just, Pythagorean, and 12-TET musical scales, the frequency ratios α for each musical scale are known. Taking $f_{j}=\alpha_{\left(j-i\right)}f_{i}$ and $f_{s}=\alpha_{\left(s-r\right)}f_{r}$, with $\alpha_{\left(j-i\right)}>1$ and $\alpha_{\left(s-r\right)}>1$. Then, Equation (16) can be written as:

(17) $$\frac{f_{i}}{f_{r}}=\frac{\alpha_{\left(s-r\right)}-1}{\alpha_{\left(j-i\right)}-1}\;\mathrm{and}\;\frac{f_{i}^{2}}{f_{r}^{2}}=\frac{\alpha_{\left(s-r\right)}^{2}-1}{\alpha_{\left(j-i\right)}^{2}-1}.$$

Then, for i>r and i<r, $f_{i}=\alpha_{\left(i-r\right)}.f_{r}$ and $f_{r}=\alpha_{\left(r-i\right)}.f_{i}$, respectively. Therefore, Equation (17) can be written for the frequency difference as:

(18) $$\frac{\alpha_{\left(s-r\right)}-1}{\alpha_{\left(j-i\right)}-1}=\alpha_{\left(i-r\right)}\;\mathrm{for}\;i>r\;\mathrm{and}\;\frac{\alpha_{\left(j-i\right)}-1}{\alpha_{\left(s-r\right)}-1}=\alpha_{\left(r-i\right)}\;\mathrm{for}\;i<r\;,$$

and for the difference between the squares of the frequencies as:

(19) $$\frac{\alpha_{\left(s-r\right)}^{2}-1}{\alpha_{\left(j-i\right)}^{2}-1}=\alpha_{\left(i-r\right)}^{2}\;\mathrm{for}\;i>r\;\mathrm{and}\;\frac{\alpha_{\left(j-i\right)}^{2}-1}{\alpha_{\left(s-r\right)}^{2}-1}=\alpha_{\left(r-i\right)}^{2}\;\mathrm{for}\;i<r\;.$$

For the frequency difference, if at least one of the equations presented in (18) is satisfied, then there are several pairs of fundamental frequencies with the same frequency difference. For the difference in the squares of the frequencies, if at least one of the equations presented in (19) is satisfied, then several pairs of fundamental frequencies have the same difference in the squares of the frequencies. We call the set of equations given in (18) and (19) the “degeneracy equations” of musical intervals.

The number of combinations of α ratios satisfying the degeneracy equations depends on the precision in the measurement of the α ratios.

Table 1 shows the number of combinations of α ratios satisfying the degeneracy equations as a function of the number of decimal places *d* used to measure these ratios (1≤d≤10). See Supplementary Note S1. Two possible situations are considered: Intervals up to two octaves, for which it is possible to interpret the quantities $f_{j}-f_{i}$ and $f_{j}^{2}-f_{i}^{2}$ as proportional to the interval sizes; and the case of all possible intervals on an 88-pitch musical instrument, such as a traditional piano.

For intervals with size *L* up to two octaves ($L_{max}=24$ semitones), the number of possible combinations of the α and $\alpha^{2}$ ratios is 24×24×23 = 13,248. For intervals with sizes up to 87 semitones (corresponding to an 88-pitch musical instrument), the number of possible combinations is 87×87×86 = 650,934.

In the 12-TET scale, the quantity $f_{j}-f_{i}$ distinguishes each pair of different pitches when the degeneracy is lifted by rounding the value of the α ratio to d≥5 for the 24 and 87 semitones cases. The quantity $f_{j}^{2}-f_{i}^{2}$ lifts the degeneracy for d≥4 in the case of 24 semitones, and for d≥8 in the case of 87 semitones (see Table 1). In the Pythagorean scale, the degeneracy of the quantity $f_{j}^{2}-f_{i}^{2}$ can only be lifted for the 24 semitones case, taking d≥5, and the degeneracy of $f_{j}-f_{i}$ cannot be lifted with up to 10 decimal places (d=10) (see Table 1). In the just scale, the degeneracy remains up to d=10 for both quantities and in both cases (24 and 87 semitones) (see Table 1).

In some cases, the degeneracy equations are satisfied independently of the precision used to measure the α ratios. For example, in the case of the quantity $f_{j}^{2}-f_{i}^{2}$ for the just scale, the combination of $\alpha_{\left(s-r\right)}=5/3$ and $\alpha_{\left(j-i\right)}=5/4$ produces $\alpha_{\left(i-r\right)}=16/9$, i.e., the major thirds (5/3) produce equal values to major sixths (5/4) when the lowest pitches of each of these intervals generate minor sevenths (16/9).

Summarizing, whenever it is possible to lift the degeneracy, this can be done through the precision of the α ratios, which depends on the precision in the measurement of the fundamental frequencies. See Supplementary Note S2.

## 3. Connection with Tonal Consonance

This section shows the connection between the representation of musical intervals previously presented and the tonal consonance formalism.

### 3.1. Measuring the Dissonance Levels of Intervals

The consonance and dissonance sensations experienced by listeners are related with the perception of pleasantness or unpleasantness produced by a combination of sounds. This sensation is fundamental in music because it is present in timbre, harmony, melody, and musical tuning [35,36,37].

The frequency difference is widely used to determine the dissonance level of a pair of pure tones sounded together [11]. In addition, this difference contains information about the interval size and its corresponding location in the register. Various models have been proposed that use the frequency difference to determine the dissonance levels of pure and complex tones sounded together [35,36,38,39,40]. One of the most recent approaches was developed by Vassilakis [39,40], which modified a model proposed by Sethares [35,38]. The model includes the dependence of the roughness on the intensity, amplitude fluctuation degree, and amplitude fluctuation rate [40]. In this model, the dissonance level δ produced by two pure tones with frequencies $f_{i}$ and $f_{j}$ and amplitudes $a_{i}$ and $a_{j}$, respectively, is given by:

(20) $$\delta =\left(0.5\right){\left[\left(a_{max}\right)\left(a_{min}\right)\right]}^{0.1}{\left[\frac{2a_{min}}{a_{max}+a_{min}}\right]}^{3.11}\left[e^{-b_{1}s\left(f_{max}-f_{min}\right)}-e^{-b_{2}s\left(f_{max}-f_{min}\right)}\right],$$

with $f_{max}=max\left(f_{i},f_{j}\right)$, $f_{min}=min\left(f_{i},f_{j}\right)$, $a_{max}=max\left(a_{i},a_{j}\right)$, $a_{min}=min\left(a_{i},a_{j}\right)$, $b_{1}=3.5$, $b_{2}=5.75$, and $s=0.24/(0.0207f_{min}+18.96)$.

Musical instruments produce complex tones composed of pure tones. The dissonance level *D* of two simultaneous complex tones with the same timbre, as in the case of a harmonic interval, can be calculated using Equation (20), taking into account the contributions of all individual dissonances δ generated from all possible combinations of pure tones in the superposition of the spectra (see Supplementary Note S3 for further details). This procedure for estimating the dissonance levels of complex tones assumes that the main contribution for the perception of the timbre comes from the spectrum [35,36,38,39], which is a reasonable assumption taking into account the fact that the timbre is strongly dependent on the spectrum and weakly dependent on the other physical parameters of the sound waves [32].

**Figure 3:**
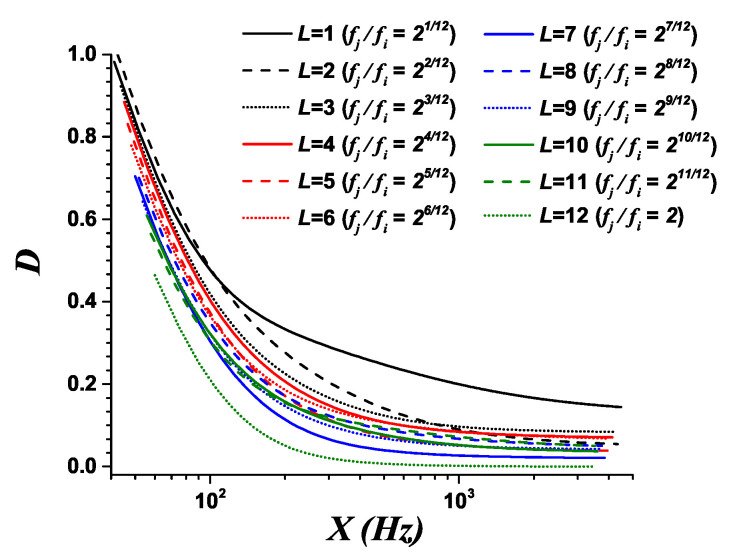
Relation between the dissonance level *D* and the locations of harmonic intervals in the register $X=(f_{j}+f_{i})/2$ for the 12-TET scale. The spectrum of each complex tone contains six harmonics with amplitudes falling at a rate of 0.88. Each possible size *L* corresponds to a particular frequency ratio inside the octave in the 12-TET scale. The dissonance level has been normalized to 1 for the typical register of an 88 key piano.

Figure 3 presents the dissonance curves for the intervals within the octave in the case of the 12-TET scale. The spectrum of each complex tone corresponds to six harmonics, with amplitudes falling at a rate of 0.88, as proposed by Sethares [35]. Explicitly, this is $A_{n}=A_{0}{\left(0.88\right)}^{n}$, where $A_{0}$ is the amplitude of the fundamental and $A_{n}$ is that of the corresponding harmonic n=1,2,3,4,5,6.

Figure 3 shows that the same interval of size *L* is less dissonant in the middle part of the register than in the lowest part, which is a well-known property of intervals [10,11].

For each interval size *L* inside the octave, the dissonance level depends on its corresponding location in the register $X=(f_{j}+f_{i})/2$. The fit to exponential functions is:

(21) $$\begin{array}{c} \begin{array}{c} D=F\left(X\right)=A_{1}exp\left(-X/\gamma_{1}\right)+A_{2}exp\left(-X/\gamma_{2}\right)+A_{3}\;, \end{array} \end{array}$$

with fit parameters $A_{1}$, $A_{2}$, $A_{3}$, $\gamma_{1}$, and $\gamma_{2}$. The values for the fit parameters of each interval size, and the corresponding determination coefficients $R^{2}$, are presented in Supplementary Table S2.

In the case of intervals larger than the octave, the *chroma* property of pitches states that the consonance values of these intervals can be measured by displacing the highest pitch to the next lower octave until the resulting interval is smaller than or equal to one octave [10]. With this property, the plots shown in Figure 3 can be employed to measure the tonal consonance levels of all possible interval sizes located at any part of the register.

### 3.2. Expected Values of the Dissonance Levels Associated to Intervals

Suppose that in a musical piece, the probability associated to the frequency of occurrence of each harmonic interval size *L* is known as $\left\{p_{{}_{L}}\right\}$ with $\sum_{L}p_{{}_{L}}=1$. The average dissonance associated to harmonic intervals can be defined as:

(22) $$\langle D\rangle =\frac{1}{H}\sum_{j}D_{j},$$

where *H* is the total number of harmonic intervals in the musical score. Grouping by intervals of equal size, as in Equation (6), we have that:

(23) $$\langle D\rangle =\frac{\sum_{i}D_{i}{|}_{{}_{L_{min}}}+\ldots +\sum_{i^{\prime }}D_{i^{\prime }}{|}_{{}_{L_{max}}}}{N}=\frac{N_{L_{min}}{\langle D\rangle }_{L_{min}}}{N}+\ldots +\frac{N_{L_{max}}{\langle D\rangle }_{L_{max}}}{N},$$

and taking $p_{{}_{L_{i}}}=N_{{}_{L_{i}}}/N$ as the probability of finding an interval of size $L_{i}$, the expected value of dissonance in a musical piece owing to the contribution of harmonic intervals is:

(24) $$\langle D\rangle =\sum_{L=L_{min}}^{L_{max}}{\langle D\rangle }_{{}_{L}}p_{{}_{L}}.$$

If all harmonic intervals have the same timbre and *D* can be expressed as in Equation (21), then the average dissonance for each kind of interval size ${\langle D\rangle }_{L}$ can be approximately obtained by expanding Equation (21) in a Taylor series around the mean position in the register (see Supplementary Note S4 for further details):

(25) $$\begin{array}{c} \begin{array}{c} {\langle D\rangle }_{L}\approx F\left({\langle X\rangle }_{L}\right)+\frac{1}{2}F^{\prime \prime }\left({\langle X\rangle }_{L}\right)\sigma_{L}^{2}\;. \end{array} \end{array}$$

The first term in Equation (25) results from the first-order approximation in the Taylor expansion, indicating that the mean location in the register for each kind of interval size ${\langle X\rangle }_{L}$ corresponds to the most important contribution to measuring the mean dissonance. The second term in Equation (25) results from the second-order term in the expansion, indicating that the dispersion of each interval size $\sigma_{L}^{2}$ is necessary to more precisely measure the mean dissonance 〈D〉.

To summarize, by knowing ${\langle X\rangle }_{L}$ and the set of probabilities $\left\{p_{{}_{L}}\right\}$, it is possible to measure L, the expected value of $\langle |f_{j}-f_{i}|\rangle$, and approximate the mean dissonance level 〈D〉. On the other hand, by knowing ${\langle X\rangle }_{L}$, $\sigma_{L}^{2}$, and $\left\{p_{{}_{L}}\right\}$, it is possible to measure L, the expected value $\langle |f_{j}^{2}-f_{i}^{2}|\rangle$, and the mean dissonance level 〈D〉 with greater precision.

Traditionally, consonance properties have been associated with simultaneous sounds. However, there is evidence of the perception of consonance also in the case of successive sounds [33,41,42]. A suitable reason for the production of consonance or dissonance sensations in melody is the short-term persistence of pitch generated by successive pitches, especially in auditoriums, and the persistence in the memory of the previous pitch [41,42]. It has been observed that musicians tend to transpose their knowledge about the consonance of harmonic intervals to judge melodic ones. These results were found in the case of isolated successive pitches [33]. Under these conditions, the consonance level of melodic intervals can be approximated using the level of consonance of harmonic ones.

## 4. Melody and Expected Values of Melodic Intervals

This section presents some concepts about melody and the expected values associated to the asymmetry in the use of ascending and descending intervals in melodic lines.

### 4.1. Concerning Melody

**Figure 4:**
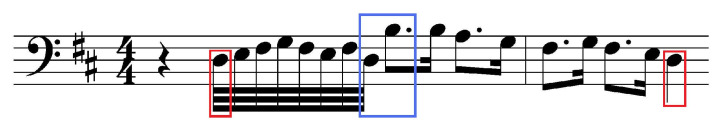
Asymmetry in the use of ascending and descending intervals in melody. Fragment from the *Fugue in D major BWV 850*, of *The Well-Tempered Clavier, Book 1* of J. S. Bach that begins and ends with the pitch *D* (red boxes), with an ascending jump (blue box) compensated using several small descending intervals.

Melody is defined in the New Grove Dictionary of Music and Musicians as “pitched sounds arranged in musical time in accordance with given cultural conventions and constraints” [43]. A definition that encompasses music and speech was given by Aniruddh Patel as “an organized sequence of pitches that conveys a rich variety of information to a listener” [31]. Melodies tend to meander around a central pitch range, and in many cultures, an asymmetry emerges, in the sense that large melodic intervals are more likely to ascend than small ones [5,44]. Figure 4 illustrates this asymmetry with a fragment extracted from the *Fugue in D major BWV 850*, of *The Well-Tempered Clavier, Book 1* of J. S. Bach. The melody begins and ends with the pitch *D* (red boxes), and the ascending jump (blue box) is compensated using small descending intervals.

So far, the sign of the interval size *L* has not been considered, as pitches in harmonic intervals are played simultaneously. However, in the case of melody, pitches are ordered chronologically (melodic intervals). For $f_{i}=f_{i}\left(t\right)$ and $f_{j}=f_{j}\left(t+1\right)$, there are three possible cases: If $f_{j}>f_{i}$, then L=j−i>0 (ascending interval), if $f_{j}<f_{i}$, then L=j−i<0 (descending interval), and if $f_{i}=f_{j}$, then L=0 (unison). Therefore, the sign of *L* distinguishes the chronological order of a pair of pitches.

For the case of the quantities $f_{j}-f_{i}$ and $f_{j}^{2}-f_{i}^{2}$, the following notation will be employed: If $\left\{t_{z}\right\}$ represents a collection of times, at each of which one pitch is played in a melody (without rests), then the quantities $f_{t_{\left(z+1\right)}}-f_{t_{z}}\equiv f_{t+1}-f_{t}$ and $f_{t_{\left(z+1\right)}}^{2}-f_{t_{z}}^{2}\equiv f_{t+1}^{2}-f_{t}^{2}$ symbolize melodic intervals, with the sign distinguishing between ascending ($f_{t+1}>f_{t}$) and descending ($f_{t+1}<f_{t}$) intervals.

The case of $f_{j}>f_{i}$ and $f_{s}>f_{r}$, which corresponds to ascending intervals, was analyzed in the section on the distinguishability of pairs of pitches. The case with $f_{j}<f_{i}$ and $f_{s}<f_{r}$, which corresponds to descending intervals, is completely equivalent (see Equation (16)).

### 4.2. Expected Values of Melodic Intervals

In the case of melody, there are three kinds of melodic intervals, ascending, descending, and unisons, and the normalization constraint may be stated as ${\tilde{p}}_{a}+{\tilde{p}}_{d}+{\tilde{p}}_{u}=1$, where ${\tilde{p}}_{a}$ is the probability of ascending intervals, ${\tilde{p}}_{d}$ is the probability of descending ones, and ${\tilde{p}}_{u}$ is the probability of unisons. The average magnitude of the melodic interval size contains the contributions of positive, negative, and zero values of *L* in the sum, $L\in [L_{min},L_{max}]$, and Equation (5) remains unaltered. The average magnitude of the melodic interval size taking into account the mean location in the register ${\langle X\rangle }_{L}$ and the dispersion $\sigma_{L}^{2}$ lead to the same expressions given previously (Equations (8) and (11)). However, now, these contain the contributions of the ascending, descending, and unison intervals. These expected values include the average magnitude of the melodic intervals but do not discriminate between ascending and descending intervals. The average magnitudes of ascending and descending intervals, $\langle L_{>0}\rangle$ and $\langle L_{<0}\rangle$, respectively, can be measured by:

(26) $$\langle L_{>0}\rangle =\frac{1}{{\tilde{p}}_{a}}\sum_{L=1}^{L_{max}}Lp_{{}_{L}};\;\langle L_{<0}\rangle =\frac{1}{{\tilde{p}}_{d}}\sum_{L=L_{min}}^{-1}Lp_{{}_{L}}\;,$$

where the ratio $p_{i}/{\tilde{p}}_{a}$ ($p_{i}/{\tilde{p}}_{d}$) refers to the probability of the occurrence of an interval of size $L_{i}$ in the ascending (descending) intervals of a musical piece.

The asymmetry in the total number of intervals is ${\tilde{p}}_{a}-{\tilde{p}}_{d}$ and the asymmetry between the average magnitudes of ascending and descending intervals can be obtained as $\langle L_{>0}\rangle +\langle L_{<0}\rangle$, where $\langle L_{<0}\rangle <0$. Because the existing literature reports that in many cultures, large melodic intervals are more likely to ascend than small ones and that melodies tend to meander around a central pitch range [5,44], the quantity ${\tilde{p}}_{a}-{\tilde{p}}_{d}$ is expected to be negative, and the quantity $\langle L_{>0}\rangle +\langle L_{<0}\rangle$ is expected to be positive, for melodic lines of several musical pieces. See Figure 4.

The asymmetry in the average magnitudes of ascending and descending intervals, taking into account the mean position in the register ${\langle X\rangle }_{L}$ and the dispersion of the intervals $\sigma_{L}^{2}$, can be measured using $\langle {\left(f_{j}-f_{i}\right)}_{>0}\rangle +\langle {\left(f_{j}-f_{i}\right)}_{<0}\rangle$ and $\langle {\left(f_{j}^{2}-f_{i}^{2}\right)}_{>0}\rangle +\langle {\left(f_{j}^{2}-f_{i}^{2}\right)}_{<0}\rangle$. These expressions take the form:

(27) $$\begin{array}{c} \begin{array}{c} \langle {\left(f_{t+1}-f_{t}\right)}_{>0}\rangle +\langle {\left(f_{t^{\prime }+1}-f_{t^{\prime }}\right)}_{<0}\rangle =\frac{1}{{\tilde{p}}_{a}}\sum_{L=1}^{L_{max}}{\langle f_{\tau +1}-f_{\tau }\rangle }_{{}_{L}}p_{{}_{L}}+\frac{1}{{\tilde{p}}_{d}}\sum_{L=L_{min}}^{-1}{\langle f_{\tau^{\prime }+1}-f_{\tau^{\prime }}\rangle }_{{}_{L}}p_{L}\\ \approx 2c\left(\frac{1}{{\tilde{p}}_{a}}\sum_{L=1}^{L_{max}}Lp_{{}_{L}}+\frac{1}{{\tilde{p}}_{d}}\sum_{L=L_{min}}^{-1}Lp_{L}\right), \end{array} \end{array}$$

and:

(28) $$\begin{array}{c} \begin{array}{c} \langle {\left(f_{t+1}^{2}-f_{t}^{2}\right)}_{>0}\rangle +\langle {\left(f_{t^{\prime }+1}^{2}-f_{t^{\prime }}^{2}\right)}_{<0}\rangle =\frac{1}{{\tilde{p}}_{a}}\sum_{L=1}^{L_{max}}{\langle f_{\tau +1}^{2}-f_{\tau }^{2}\rangle }_{{}_{L}}p_{L}+\frac{1}{{\tilde{p}}_{d}}\sum_{L=L_{min}}^{-1}{\langle f_{\tau^{\prime }+1}^{2}-f_{\tau^{\prime }}^{2}\rangle }_{{}_{L}}p_{L}\\ \approx 4c\left(\frac{1}{{\tilde{p}}_{a}}\sum_{L=1}^{L_{max}}Lp_{{}_{L}}+\frac{1}{{\tilde{p}}_{d}}\sum_{L=L_{min}}^{-1}Lp_{L}\right). \end{array} \end{array}$$

With respect to the consonance issue, assuming for practical purposes that the results found for the dissonance level of isolated melodic intervals, in the case of musicians, can be used inside a melody, the mean dissonance level associated to a melodic line could be measured using (24), taking into account the contributions of ascending and descending intervals, and melodic unisons. For the consonance analysis of melodic intervals, the sign of *L* is irrelevant: Only its magnitude is important. Then, Figure 3 can be utilized for ascending intervals as well as descending ones.

Up to now, we have developed a representation of musical intervals suitable for analyzing harmony as well as melody. From now on, we limit the analysis to melody.

## 5. Materials and Methods: An Application to Melodic Lines

This section shows the analysis of a set of melodic lines using the representation of musical intervals proposed, and the procedures followed to obtain their corresponding probability and cumulative distributions.

### 5.1. Selection of Melodic Lines

Twenty melodic lines from seven vocal and instrumental masterpieces of the Baroque and Classical periods were analyzed. The selected pieces contain melodic lines characterized by their considerable length, internal coherence, and rich variety of instruments and registers. The collection of pieces is as follows:

- *Brandenburg Concerto No. 3 in G Major BWV 1048*. Johann Sebastian Bach: Polyphonic concerto for 11 musical instruments (three violins, three violas, three cellos, *violone*, and harpsichord).
- *Missa Super Dixit Maria*. Hans Leo Hassler: Polyphonic composition for four voices (soprano, contralto, tenor, and bass).
- *First movement of the Partita in A Minor BWV 1013*. Johann Sebastian Bach: This piece has just one melodic line for a flute.
- *Piccolo Concerto RV444*. Antonio Vivaldi (arrangement by Gustav Anderson): We selected the piccolo melodic line, owing to its rich melodic content.
- *Sonata KV 545*. Wolfgang Amadeus Mozart: We selected the melodic line for the right hand of this piano sonata, assuming that it drives the melodic content.
- *Suite No. 1 in G Major BWV 1007* and *Suite No. 2 in D Minor BWV 1008*. Johann Sebastian Bach: The melodic lines of these pieces written for cello contain mainly successive pitches. In the cases of the few simultaneous pitches, the continuation of the melodic lines was assumed in the direction of the highest pitch.

### 5.2. Procedure to Obtain the Probability and the Cumulative Distributions

The PDs for the quantities $f_{t+1}-f_{t}$ and $f_{t+1}^{2}-f_{t}^{2}$ were obtained for each melodic line in order to gather information concerning the selections of melodic intervals made by the composers. The procedure for the analysis of melodic lines was as follows:

- The MIDI files were generated from scores. Only successive pitches without rests between them were considered.
- The MIDI information was transformed into frequencies using the 12-TET scale with A=440 Hz. Supplementary Spreadsheet S1 contains the data $f_{t}$ and $f_{t+1}$ in Hz, corresponding to the melodic intervals of each melodic line.
- The PDs were obtained in three different cases:
  - Case 1: $|f_{t+1}-f_{t}|$ and $|f_{t+1}^{2}-f_{t}^{2}|$ not distinguishing between ascending and descending intervals. The complementary cumulative distribution (CCD) was also obtained.
  - Case 2: $|f_{t+1}-f_{t}|$ and $|f_{t+1}^{2}-f_{t}^{2}|$ for two different sets of intervals: Ascending and unisons, and descending and unisons. The CCD was also obtained for each set.
  - Case 3: $f_{t+1}^{2}-f_{t}^{2}$ for the set of ascending, descending, and unison intervals together. In this case, the sign of the descending intervals was considered as negative. The reason for only using the quantity $f_{t+1}^{2}-f_{t}^{2}$ is the quality of the experimental fits obtained in the two previous analyses for both quantities, and even more relevantly that the distinguishability analysis shows that $f_{t+1}^{2}-f_{t}^{2}$ has the best resolution properties for the case of 24 semitones in the 12-TET scale (see Table 1), which is the relevant range for melodic intervals in the analyzed melodic lines. The CCD was employed for the branch of the PD that contains the ascending intervals, and the cumulative distribution (CD) was utilized for the branch that contains the descending intervals.

Some clarifications are required in order to implement the sketch described above:

- Because the number of melodic intervals in the studied melodic lines is at most one order of magnitude larger than the total number of possible pairs of successive pitches generated by the same *ambitus* (the range between the lowest and highest pitches) of the original melodic line, the PDs were constructed using histograms, in order to capture significant probabilities. Supplementary Table S3 shows the number of intervals of each melodic line, the number of ascending intervals, descending ones, and unisons, and the corresponding *ambitus*.
- As the number of possible melodic intervals for any melodic line is finite, independently of its length, the bin width in the histograms will be moderately dependent on the number of melodic intervals. This condition is satisfied by the Sturges criterion [45], and thus, this criterion was used to determine the bin width.
- In the third case, when ascending and descending PDs were combined in the same distribution for the quantity $f_{t+1}^{2}-f_{t}^{2}$, the bin width was taken as the average of those obtained separately using the Sturges criterion for ascending and descending distributions. The average bins were symmetrically located to the left and right, starting from the point $f_{t+1}^{2}-f_{t}^{2}=0$.
- In the experimental analysis, the contribution of unisons in the histograms is important for ascending intervals as well as descending ones, with different right-hand and left-hand limits at 0. In addition, if we attempt to split the unisons into the ascending and descending parts, this procedure reduces the determination coefficient $R^{2}$ of the fits for the histograms to an exponential function [46]. Hence, all unisons were included in the ascending part as well as the descending one, and then a correction of this double count was carried out in the procedure to obtain the expected values. In the histograms, the descending intervals are contained inside the bins labeled from 1 to N/2 (from left to right), and the ascending ones inside those labeled from N/2+1 to *N* (from left to right). Hence, all unisons have been taken into account inside the bin labeled N/2 as well as that labelled N/2+1. Notice that *N* is an even number.

## 6. Results and Discussion

This section shows the experimental probability and cumulative distributions of the studied melodic lines, the Shannon entropy of intervals of two successive pitches in melodic lines, a statistical model based on the minimization of the Kullback–Leibler divergence that reproduces the main features of the experimental results, and the connection between the parameters of the statistical model with the transposition processes, the asymmetry between ascending and descending intervals, and the mean dissonance level of the studied melodic lines.

### 6.1. Experimental Results and Analysis

For the first and the second cases, the histograms and CCD for both quantities ($|f_{t+1}-f_{t}|$ and $|f_{t+1}^{2}-f_{t}^{2}|$) fit to exponential functions. Supplementary Table S4 shows, for each melodic line in the first and the second case, the determination coefficient $R^{2}$ for the fits to exponential functions in histograms and CCD. The average $\bar{R^{2}}$ of the CCD is $\bar{R^{2}}\approx 0.99$, with a standard deviation (SD) of ≈0.01. Usually, the cumulative probability associated to the unison in the CCD is larger than the value predicted by the exponential behavior. This is not surprising, as the value 0 is degenerated and represents more than one possible pair of pitches. For histograms, the highest $\bar{R^{2}}$ is for the quantity $|f_{t+1}^{2}-f_{t}^{2}|$, with ascending and descending intervals taken separately. For ascending intervals, $\bar{R^{2}}=0.987$ with SD=0.009, and for descending ones $\bar{R^{2}}=0.986$ with SD=0.016.

**Figure 5:**
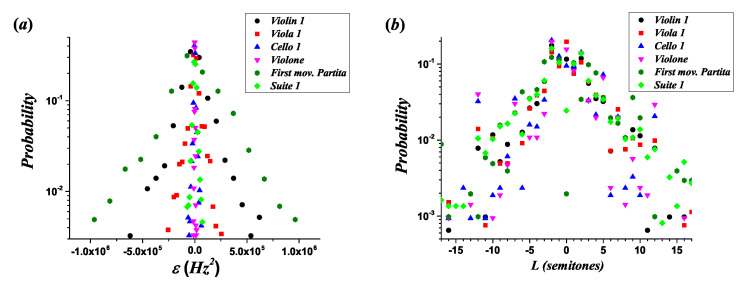
Probability distributions of melodic intervals for the following melodic lines: Violin 1, viola 1, cello 1, and *violone* from the *Brandenburg Concerto No. 3 in G Major BWV 1048*, the *first movement of the Partita in A Minor BWV 1013*, and the *Suite No. 1 in G Major BWV 1007*. (**a**) Quantity $f_{t+1}^{2}-f_{t}^{2}$ measure using bins (ε). (**b**) Traditional melodic interval size *L* in semitones.

For the third case, with the left and right branches of the PD combined in the same histogram, the PD can be written as:

(29) $$P\left(\varepsilon \right)=\left\{\begin{array}{cc} F_{+}^{H}e^{-\varepsilon /G_{+}^{H}} & \mathrm{for}\;\varepsilon >0\\ F_{-}^{H}e^{\varepsilon /G_{-}^{H}} & \mathrm{for}\;\varepsilon <0 \end{array}\right.,$$

where the notation ε emphasizes that these distributions are constructed over bins. Figure 5 shows a set of probability distributions of melodic intervals for the quantity ε, panel (a), and for the melodic interval size *L* measured in semitones, panel (b). Notice that the traditional interval size does not distinguish the register of the musical instruments.

In the case of the cumulative distributions, the CCD and CD conserve the same functional form of the PD (as the PDs are exponential):

(30) $$\begin{array}{c} \begin{array}{c} P\left(f_{t+1}^{2}-f_{t}^{2}\right)=\left\{\begin{array}{cc} F_{+}^{C}e^{-(f_{t+1}^{2}-f_{t}^{2})}/G_{+}^{C} & \mathrm{for}\;(f_{t+1}^{2}-f_{t}^{2})>0\\ F_{-}^{C}e^{\left(f_{t+1}^{2}-f_{t}^{2}\right)}/G_{-}^{C} & \mathrm{for}\;(f_{t+1}^{2}-f_{t}^{2})<0 \end{array}\right.. \end{array} \end{array}$$

**Figure 6:**
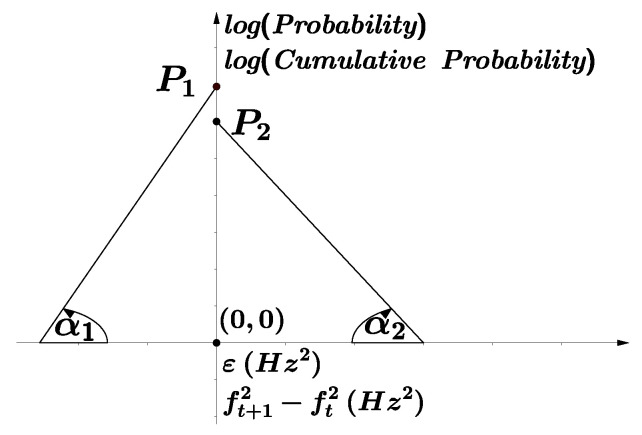
General forms of the probability and cumulative distributions P(ε) and $P(f_{t+1}^{2}-f_{t}^{2})$, respectively. In the symmetric case, $P_{1}=P_{2}$ and $\alpha_{1}=\alpha_{2}$.

Supplementary Table S5 contains the values of $F_{+}^{H},F_{-}^{H},G_{+}^{H},G_{-}^{H},F_{+}^{C},F_{-}^{C},G_{+}^{C},G_{-}^{C}$, and $R^{2}$ for the fits. These PDs resemble the asymmetric Laplace PD, with different amplitudes for positive and negative branches leading to a discontinuity at the origin (Figure 6) [47].

**Figure 7:**
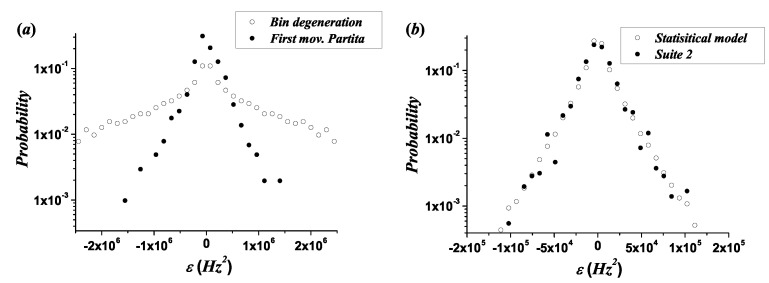
(**a**) Comparison between the Probability distributions (PDs) for the real melodic line of the first movement of the *Partita in A minor BWV 1013* by J. S. Bach and for the corresponding bin degeneration for the same *ambitus*. (**b**) Comparison between histogram for the melodic line of *Suite No. 2 BWV 1008* by J. S. Bach and that produced by the statistical model.

Figure 7a shows the histogram of the PD for the first movement of the *Partita in A minor BWV 1013*, as well as the PD for the bin degeneration in the corresponding *ambitus*, which originates from the structure of the musical scale and represents the melodic line with the highest diversity of melodic intervals in different locations of the register. The bin degeneration PD is equivalent to that of a long random melodic line (see Supplementary Note S5 for further details). In order to explain the effect of bin degeneration, notice that the distance in $Hz^{2}$ between pairs of differences $f_{j}^{2}-f_{i}^{2}$ for the 12-TET scale varies in such a manner that the number of differences inside an arbitrary bin ε, representing its degeneracy, decreases when $|f_{j}^{2}-f_{i}^{2}|$ increases.

The comparison between the distributions of real melodic lines and those from bin degeneration for the corresponding *ambitus* indicates that the scale contributes to the observed results but does not explain them. In addition, the PD for bin degeneration fits better to a power law function ($\bar{R^{2}}=0.963$) than to an exponential function ($\bar{R^{2}}=0.934$). Supplementary Table S6 contains the determination coefficient $R_{2}$ for the fit to a power law and an exponential function, in the case of each melodic line.

The quantitative difference between the PD for a real melodic line and its corresponding random one (the bin degeneration PD) provides information on the order introduced into the system by the composer, stemming from the selection of successive pairs of pitches. A mathematical tool for comparing two PDs is provided by the Kullback–Leibler divergence, or relative entropy [48]:

(31) $$D_{KL}=\sum_{k=1}^{N}p_{k}\ln\left(\frac{p_{k}}{q_{k}}\right),$$

where $p_{k}$ is the PD for the real melodic line to be compared with the a priori distribution $q_{k}$ coming from the degeneration of the $k^{th}$ bin, and *N* is the number of bins in the *ambitus* with N/2 bins for each branch (ascending and descending). The PD $q_{k}$ has been formally related to the probability associated with the number of distinguishable subcategories in the category *k*, representing its degeneracy [49].

The minimization of the relative entropy under constraints is useful to describe the form of the PD, as is explained in the next section.

### 6.2. Shannon Entropy of Melodic Intervals in Melodic Lines

Assuming that each possible melodic interval generated from the *ambitus* of a melodic line corresponds to a possible state, an analysis of the evolution of the entropy of melodic intervals in the progression of the melodic line can be performed in a similar manner as in the work by G. Gündüz and U. Gündüz [28]. For the A different pitches inside the *ambitus* of a melodic line, the number of different melodic intervals is $A^{2}$. Following [28], we used the Shannon entropy:

(32) $$S\;\left(bits\right)=-\sum_{m=1}^{M}p_{m}log_{2}p_{m}\;,$$

where *M* refers to the final melodic interval appearing in the progression of the melodic line, and $p_{m}$ is the probability that the interval *m* has already appeared in the sequence. The final Shannon entropy $S_{f}$ is reached when *M* is equal to the total number of melodic intervals in the melodic line.

**Figure 8:**
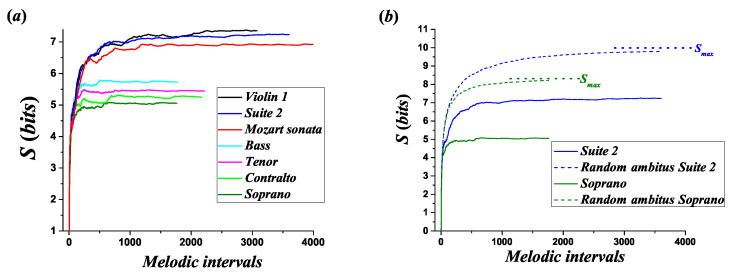
(**a**) Evolution of the Shannon entropy of melodic intervals for different melodic lines. (**b**) Evolution of the Shannon entropy of melodic intervals for the melodic lines of the soprano, in the *Missa Super Dixit Maria*, and *Suite 2 BWV 1008* with the corresponding random melodies constructed using the same *ambitus*. The maximum Shannon entropy of melodic intervals $S_{max}$ corresponds to the maximum possible value of the Shannon entropy of melodic intervals in a long random melodic line with the same *ambitus* as the original one.

Figure 8 illustrates the evolution of the Shannon entropy of melodic intervals in melodic lines, from now on entropy. Panel (a) shows several melodic lines. Panel (b) shows the melodic lines of the *Suite No. 2 BWV 1008*, and the soprano in the *Missa Super Dixit Maria*, with their corresponding random melodies constructed using the same *ambitus*. The maximum entropy $S_{max}$ corresponds to the maximum possible value of the entropy in a long random melodic line with the same *ambitus* as the original one, namely $S_{max}=log_{2}\left(A^{2}\right)$.

Figure 8a,b shows that the entropy increases with each new melodic interval in the progression until it reaches a limiting value, which is smaller than the entropy of a random melodic line with the same *ambitus*. Some fluctuations appear in this process. However, the entropy tends to be stabilized at the final section of the melodic line. This result is similar to the findings of G. Gündüz and U. Gündüz analyzing the entropy evolution associated to the connectivity of pitches in different melodies [28].

For each melodic line, Table 2 presents the final entropy $S_{f}$, the maximum entropy reached by the melodic line $S_{max}^{*}$, and the maximum entropy generated by the *ambitus* of the corresponding melodic line $S_{max}$.

### 6.3. Statistical Model for Melodic Lines: Relative Entropy Minimization under Macroscopic Constraints

From the previously presented definitions of melody [31,43] and the results in Figure 8a,b and Table 2, we infer that the composer creates a melodic line among the richest in terms of the use of melodic intervals, but in accordance with musical constraints. Because each melodic interval in the 12-TET scale corresponds to a particular value of $f_{t+1}^{2}-f_{t}^{2}$ (except for unisons), and the expected value of this quantity contains musical information, the work carried out by the composer can be modeled as a procedure in which the relative entropy is minimized (the closest $p_{k}$ to $q_{k}$) under constraints with musical meaning.

Different musical constraints can be proposed in order to reduce the entropy value of a melodic line away from that of a random one, and we propose the following ones.

Assuming that the total numbers of ascending and descending intervals and unisons are known, the first two constraints measured from histograms are:

(33) $${\tilde{p}}_{d}+{\tilde{p}}_{u}=\sum_{k=1}^{N/2}p_{k}\;\mathrm{and}\;{\tilde{p}}_{a}+{\tilde{p}}_{u}=\sum_{k=(\frac{N}{2}+1)}^{N}p_{k}\;,$$

where ${\tilde{p}}_{a}$ is the probability of an ascending interval, ${\tilde{p}}_{d}$ is that of a descending one, ${\tilde{p}}_{u}$ is the probability of a unison, and ${\tilde{p}}_{a}+{\tilde{p}}_{d}+{\tilde{p}}_{u}=1$. Here, the unisons contribute to the ascending part as well as the descending part, as was explained in the methods section.

The next constraint comes from the best estimation of the average magnitude of the melodic intervals using histograms (Equation (11)):

(34) $$\begin{array}{c} \begin{array}{c} \langle |\varepsilon |\rangle =\sum_{k=1}^{N}p_{k}\cdot |\varepsilon_{k}|-\frac{1}{2}[{\tilde{p}}_{u}|\varepsilon_{N/2}|+{\tilde{p}}_{u}|\varepsilon_{(N/2)+1}\left|\right]=\sum_{k=1}^{N}p_{k}\cdot |\varepsilon_{k}|-{\tilde{p}}_{u}\left|\varepsilon_{N/2}\right|, \end{array} \end{array}$$

where the quantity $-{\tilde{p}}_{u}\left|\varepsilon_{N/2}\right|$ corrects the double counting of unisons.

The asymmetry in the magnitudes of ascending and descending intervals is the final constraint. This asymmetry is present in the difference between the coefficients for the left and right branches in Equations (29) and (30). Using histograms, the best estimate that we can obtain for Equation (28) is:

(35) $$\begin{array}{c} \begin{array}{c} \langle \varepsilon_{>0}\;\rangle +\langle \varepsilon_{<0}\rangle =\frac{1}{{\tilde{p}}_{d}}\sum_{k=1}^{N/2}p_{k}\varepsilon_{k}+\frac{1}{{\tilde{p}}_{a}}\sum_{k=N/2+1}^{N}p_{k}\varepsilon_{k}+\left|\varepsilon_{N/2}\right|\left(\frac{{\tilde{p}}_{u}}{{\tilde{p}}_{d}}-\frac{{\tilde{p}}_{u}}{{\tilde{p}}_{a}}\right), \end{array} \end{array}$$

where the quantity $|\varepsilon_{N/2}|\left(\frac{{\tilde{p}}_{u}}{{\tilde{p}}_{d}}-\frac{{\tilde{p}}_{u}}{{\tilde{p}}_{a}}\right)$ removes the contribution of unisons.

Supplementary Table S7 contains the values of the quantities shown in Equations (11) and (28) and their corresponding approximations using histograms through Equations (34) and (35).

Minimizing the relative entropy subject to Equations (33)–(35) (in a similar procedure to that shown in [47]) produces the following PD (see Supplementary Note S6 for further details):

(36) $$\begin{array}{c} \begin{array}{c} p_{k}=\left\{\begin{array}{cc} \frac{\left({\tilde{p}}_{d}+{\tilde{p}}_{u}\right)q_{k}e^{\left(-\lambda_{1}\left|\varepsilon_{k}\right|-\frac{\lambda_{2}}{{\tilde{p}}_{d}}\varepsilon_{k}\right)}}{\sum_{m=1}^{N/2}\left[q_{m}e^{\left(-\lambda_{1}\left|\varepsilon_{m}\right|-\frac{\lambda_{2}}{{\tilde{p}}_{d}}\varepsilon_{m}\right)}\right]} & \mathrm{for}\;k\in [1,N/2]\\ \frac{\left({\tilde{p}}_{a}+{\tilde{p}}_{u}\right)q_{k}e^{\left(-\lambda_{1}\left|\varepsilon_{k}\right|-\frac{\lambda_{2}}{{\tilde{p}}_{a}}\varepsilon_{k}\right)}}{\sum_{m=\frac{N}{2}+1}^{N}\left[q_{m}e^{\left(-\lambda_{1}\left|\varepsilon_{m}\right|-\frac{\lambda_{2}}{{\tilde{p}}_{a}}\varepsilon_{m}\right)}\right]} & \mathrm{for}\;k\in [\frac{N}{2}+1,N], \end{array}\right. \end{array} \end{array}$$

where $\lambda_{1}$ and $\lambda_{2}$ are the Lagrange multipliers for Equations (34) and (35), respectively. The values of $\lambda_{1}$ and $\lambda_{2}$ were obtained using the expected values 〈|ε|〉 and 〈ε〉 from the histograms of the empirical distributions for the selected melodic lines, and allowing the relative error between the expected values from the statistical model and those from the real data to be smaller than 1.0%. Supplementary Table S7 contains the expected values used in the statistical model, and Table 2 presents the values of the Lagrange multipliers generated from them. While the values of $\lambda_{1}$ are positive, those of $\lambda_{2}$ can be positive or negative, exhibiting possible asymmetries in the use of ascending and descending intervals. In addition, $\lambda_{1}$ is between one and two orders of magnitude larger than $\lambda_{2}$.

Figure 7b presents a comparison between the statistical model and the empirical results in the case of *Suite No. 2 BWV 1008*. Some differences between the empirical data and the results from the statistical model are expected, because there are patterns in real melodic lines that cannot be captured by this simple model.

**Figure 9:**
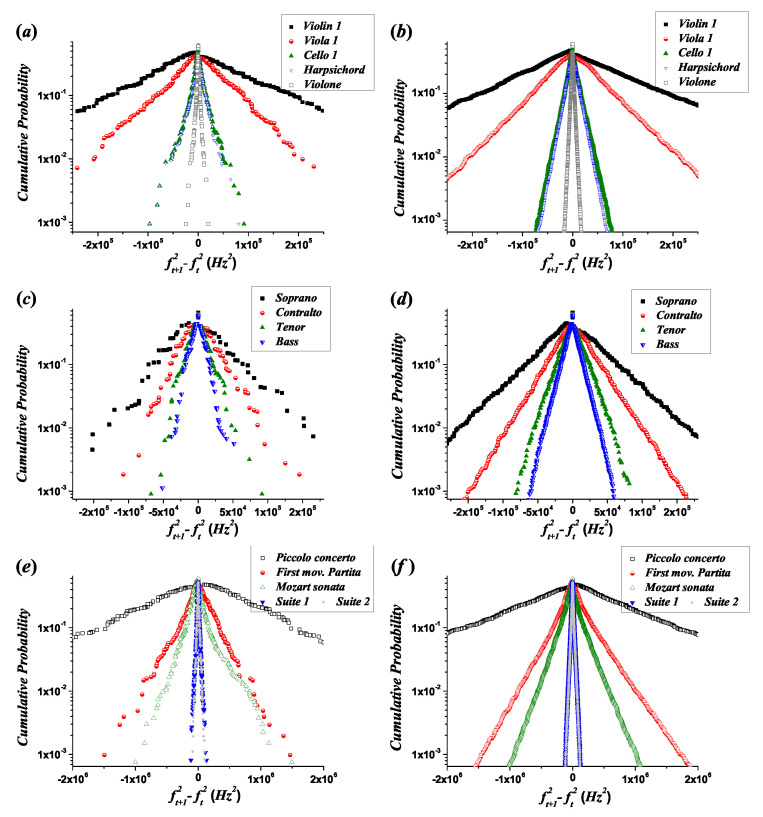
Complementary cumulative distribution (CCD) (ascending branches) and cumulative distribution (CD) (descending branches) for the empirical distributions (**a**,**c**,**e**) and the corresponding statistical model results (**b**,**d**,**f**). (**a**,**b**) *Brandenburg Concerto No. 3 in G Major BWV 1048* by J. S. Bach, (**c**,**d**) *Missa Super Dixit Maria* by Hans Leo Hassler, and (**e**,**f**) *Piccolo Concerto RV444* by Antonio Vivaldi; *First movement of the Partita in A Minor BWV 1013* by J. S. Bach; *Sonata KV 545* by W. A. Mozart; *Suite No. 1 in G Major BWV 1007* by J. S. Bach and *Suite No. 2 in D Minor BWV 1008* by J. S. Bach.

The CCD (ascending branch) and CD (descending branch) can be utilized to compare different melodic lines that are either experimental or obtained from the statistical model. The CCD and CD were obtained from the histograms produced by the statistical model, randomly distributing the probability assigned to a bin between all the possible melodic intervals inside it, which were generated using the *ambitus* of the corresponding melodic line. Because ${\tilde{p}}_{u}$ is known, the probability assigned to 0 inside the bins containing unisons was taken as ${\tilde{p}}_{u}$, and the remaining probability of the bin was distributed randomly in the other possible melodic intervals. Figure 9 depicts the CCD and CD for the empirical data and the corresponding results from the statistical model for most melodic lines. In this figure, and taking into account the values in Table 2, the following features can be inferred:

1. Different registers of musical instruments and human voices can be distinguished using the Lagrange multiplier $\lambda_{1}$, allowing, for example, to discriminate between the same melodic line played in different parts of the register (a transposition). An example of a transposition is given in the *Brandenburg Concerto No. 3 BWV 1048* by J. S. Bach, in which the harpsichord plays the same melodic line as the *violone* but transposed one octave higher (the fundamental frequency ratio of the transposition is equal to 2): While the entropy evolution in these melodic lines is the same, there is a change in the exponential decay parameters, characterized by the values of the Lagrange multipliers (see Table 2), and the numerical values of the expected values are related as:
  (37) $$\begin{array}{c} {\langle |\varepsilon |\rangle }_{Harpsichord}=2^{2}{\langle |\varepsilon |\rangle }_{Violone}\\ \langle |f_{t+1}^{2}-f_{t}^{2}{|\rangle }_{Harpsichord}=2^{2}\langle |f_{t+1}^{2}-f_{t}^{2}{|\rangle }_{Violone}\\ {\left[\langle \varepsilon_{>0}\;\rangle +\langle \varepsilon_{<0}\rangle \right]}_{Harpsichord}=2^{2}{\left[\langle \varepsilon_{>0}\;\rangle +\langle \varepsilon_{<0}\rangle \right]}_{Violone}\\ {\left[\langle {\left(f_{t+1}^{2}-f_{t}^{2}\right)}_{>0}\rangle +\langle {\left(f_{t^{\prime }+1}^{2}-f_{t^{\prime }}^{2}\right)}_{<0}\rangle \right]}_{Harpsichord}=2^{2}{\left[\langle {\left(f_{t+1}^{2}-f_{t}^{2}\right)}_{>0}\rangle +\langle {\left(f_{t^{\prime }+1}^{2}-f_{t^{\prime }}^{2}\right)}_{<0}\rangle \right]}_{Violone}, \end{array}$$
  in agreement with the properties derived above for transposition processes (Equation (15)).
2. With respect to the quantitative results of the model, the orders of magnitude of the fit parameters of the statistical model are in agreement with the corresponding results of the experimental fits. For each melodic line, Supplementary Table S8 contains the fit parameters to discontinuous asymmetric Laplace distributions, generated from the statistical model results. The average relative error in the histograms for the amplitude of the exponential distributions is 17.1%, and that for the decay coefficient is 20.6%. In the cases of the CD and CCD, the average errors of the amplitude and the decay coefficient are 7.2% and 11.8%, respectively. Supplementary Table S9 contains the values of these errors for each melodic line.
3. In most cases (90% of the melodic lines), Equation (35) takes positive values (corresponding to negative values of $\lambda_{2}$), and ${\tilde{p}}_{a}-{\tilde{p}}_{d}$ takes negative values (see Supplementary Table S3). This behavior is consistent with the asymmetry represented in Figure 4, in the sense that the magnitudes of ascending intervals are expected to be larger than those of descending ones, and the total number of descending intervals must be larger than that of ascending ones. Negative values of ${\tilde{p}}_{a}-{\tilde{p}}_{d}$ and $\lambda_{2}$ lead to different decay coefficients and different intercept points with the ordinate axis for the ascending and descending branches, which can be observed in the experimental fits of the CD and CCD through the comparison of the corresponding coefficients, $F_{+}^{C}<F_{-}^{C}$ and $G_{+}^{C}>G_{-}^{C}$ (see Supplementary Table S5). Figure 6 was created with the purpose of magnifying these particular asymmetries: $P_{1}>P_{2}$ and $\alpha_{1}>\alpha_{2}$ (implying that $\lambda_{2}<0$). The two exceptions are the *Piccolo Concerto RV444* of Antonio Vivaldi, where $\lambda_{2}>0$ and ${\tilde{p}}_{a}-{\tilde{p}}_{d}>0$, and the melodic line of the tenor voice in *Missa Super Dixit Maria*, where $\lambda_{2}>0$ and ${\tilde{p}}_{a}-{\tilde{p}}_{d}<0$.
4. Because the difference between $\lambda_{1}$ and $\lambda_{2}$ is between one and two orders of magnitude (i.e., the decay coefficients have the same order of magnitude), and the bin width selection affects the measure of the decay parameters, the asymmetry in the values of the decay coefficients is better observed in the cumulative distributions than in the histograms.
5. Because in Figure 6, the limit $P_{1}$ of the CD (constructed for descending intervals) when $f_{t+1}^{2}-f_{t}^{2}\to 0^{-}$ represents the probability of a value slightly smaller than 0, and in the CCD (constructed for ascending intervals), $P_{2}$ when $f_{t+1}^{2}-f_{t}^{2}\to 0^{+}$ represents the probability of a value slightly larger than 0, the asymmetry ${\tilde{p}}_{a}-{\tilde{p}}_{d}\approx P_{2}-P_{1}$. This result can be observed in Figure 9 and represents the difference in the amplitudes of the exponential decay for the CD and CCD. In most cases, except for the *Piccolo Concerto RV444*, ${\tilde{p}}_{a}<{\tilde{p}}_{d}$, implying that $P_{1}>P_{2}$. In the case of the *Piccolo Concerto RV444*, it holds that ${\tilde{p}}_{a}>{\tilde{p}}_{d}$, implying that $P_{1}<P_{2}$.

### 6.4. Transposition Processes and Mean Dissonance Level of Melodic Lines

As explained in the section on melody, tonal consonance properties can be formally associated to melodic intervals in the case of musicians. Because the musical instruments analyzed in this study use vibrating strings and air columns, the main consonance properties may be captured using the model of the harmonic spectrum presented in the tonal consonance section.

For each melodic line, the mean dissonance level 〈D〉 was measured using the curves shown in Figure 3 for intervals inside the octave, and the *chroma* properties of pitch for intervals wider than one octave. Table 2 lists the values of the mean dissonance 〈D〉 and their corresponding approximations ${\langle D\rangle }^{*}$ using ${\langle X\rangle }_{L}$ and $\sigma_{L}^{2}$ in Equation (25). Comparing ${\langle D\rangle }^{*}$ with 〈D〉, the observed relative error is less than 1.0% for all melodic lines.

From the results in Table 2, melodic lines tend to be more dissonant for instruments with lower registers, which is a well-known phenomenon in music theory [10]. An interesting case is that of transposition, as the same melodic lines played in different parts of the register have different dissonance levels. For example, the melodic line of the *violone* in the *Brandenburg Concerto BWV 1048* is perceived as more dissonant than that of the harpsichord.

**Figure 10:**
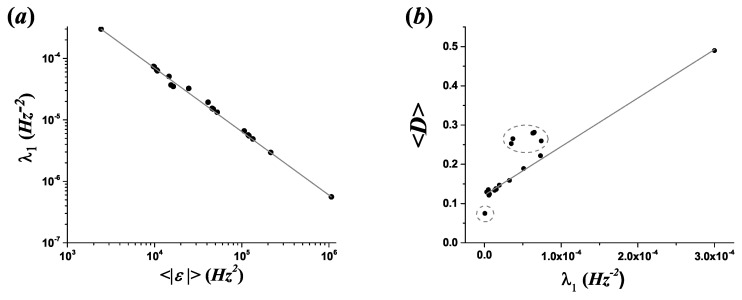
(**a**) Power law relation between the quantity 〈|ε|〉 and the Lagrange multiplier $\lambda_{1}$. (**b**) Relation between the mean dissonance 〈D〉 and the Lagrange multiplier $\lambda_{1}$. For 13 of the 20 melodic lines, a linear relation was observed.

Low registers are associated with small values of L, and therefore of $\langle |f_{t+1}^{2}-f_{t}^{2}|\rangle$ and consequently also 〈|ε|〉. For all melodic lines, a power law relation was observed between the quantity 〈|ε|〉 and the Lagrange multiplier $\lambda_{1}$ (see Figure 10a):

(38) $$\lambda_{1}=A{\langle |\varepsilon |\rangle }^{B},$$

where the magnitude of A is $9.423\times 10^{-1}\pm \left(9.76\times 10^{-2}\right)$, and $B=-1.033\pm (1.26\times 10^{-2})$, with $R^{2}=0.998$. If B is taken as −1, then A is dimensionless. Low values of 〈|ε|〉 correspond to high values of $\lambda_{1}$, and vice versa, and $\lambda_{1}$ scales in a transposition process as:

(39) $$\lambda_{1}^{N}\approx \omega^{2B}\lambda_{1}^{O}\;,$$

where $\lambda_{1}^{O}$ and $\lambda_{1}^{N}$ denote the first Lagrange multiplier in the original and new locations of the register, respectively. For the transposition between the *violone* and the harpsichord, $\lambda_{1}^{Harpsichord}\approx \omega^{2(-1.033)}\lambda_{1}^{Violone}$, with a 3% relative error (see Table 2).

For 13 of the melodic lines studied, a linear relation was observed between the mean dissonance levels of melodic lines and the first Lagrange multiplier (see Figure 10b):

(40) $$\langle D\rangle =C+D\lambda_{1}\;,$$

where $C=1.122\times 10^{-1}\pm \left(1.7\times 10^{-3}\right)$ and $D=\left(1236.29\pm 19.81\right)\;Hz^{2}$, with $R^{2}=0.997$.

The Lagrange multiplier $\lambda_{1}$ locates the approximate region of exponential decay, and for these 13 melodic lines, this geometrical parameter can be employed as an indicator of the mean dissonance properties. Strong exponential decays correspond to low registers with high dissonance levels, and vice versa. The seven pieces that do not follow a linear relation (marked with “⋆” in Table 2 and dot circles in Figure 10b) correspond to five cellos and a harpsichord, characterized by mean dissonance values between 0.25 and 0.30, and the piccolo of *Concerto RV444* with a mean dissonance level of 0.0749.

The results show that the model proposed is suitable for the classification and generation of music. In the case of melody, the expected values can be used to classify melodic lines by their location in the register and the asymmetry in the use of ascending and descending intervals. For music generation, the expected values and the relevant PDs can be used as constraints for a melodic line, which is equivalent to providing full sets of intervals to be used in the musical piece.

## 7. Conclusions

The concept of the musical interval size was extended using two physical quantities: The difference between the fundamental frequencies of pitches and the difference in the squares of the fundamental frequencies. We explored the characteristics of these quantities in three different musical scales: The just, Pythagorean, and 12-TET. We found that both quantities contain information on the size of the interval and its location in the register, owing to the existence of a relationship between the construction rules of the scales and the sizes of intervals, which becomes linear in the most relevant regime for utilization in music. These quantities can be measured with different precision levels, allowing us in many cases to lift a degeneracy associated with the traditional musical interval size concept, in the sense that it cannot distinguish intervals of the same size located in different locations of the register.

The expected values of the two physical quantities were shown to be macroscopic quantities that contain relevant musical information. Specifically, they correspond to a generalization of the traditional mean musical interval size, as the expected values also take into account the mean location and the dispersion of the intervals in the register.

A link between the theory of tonal consonance and the expected values of the two considered physical quantities was developed. Specifically, knowing the mean location of musical intervals with a given size in the register, and the corresponding variance, it is possible to measure both the expected values and the mean dissonance properties of a musical piece, owing to the use of musical intervals produced by an instrument with a particular timbre.

In order to verify the usefulness of this formalism, it was applied to melodies. The frequency of occurrences of melodic intervals in 20 melodic lines from seven masterpieces of Western tonal music was measured, and the probability distributions of both quantities were obtained. In all cases, we obtained noncontinuous asymmetric Laplace distributions. In addition, the Shannon entropy associated with the appearances of melodic intervals during the progression of a melodic line increases up to a limiting value, which is smaller than the corresponding entropy for a random composition. In order to explain these empirical findings, a statistical model based on the minimization of the relative entropy under constraints was proposed for the difference in the squares of the fundamental frequencies. Two constraints are associated with the number of ascending, descending, and unison intervals, and the two other constraints correspond to expected values arising from the average magnitude of the physical quantity, and the asymmetry in the magnitudes of ascending and descending intervals. The model includes two Lagrange multipliers. The first locates the region in the register where the melody is played, giving information on musical processes such as transposition. The second captures asymmetry patterns between ascending and descending intervals. For 13 of the 20 studied melodic lines, the first Lagrange multiplier is related to the mean dissonance level of the melodic line, connecting macroscopic statistical properties with psychoacoustic features of the system.

The presented findings show that for the studied musical pieces, the selection of melodic intervals made by the composers, including their locations in the register, can be modeled as a tight compromise between order and disorder, with a principle of entropy extremalization constrained by macroscopic quantities with musical meanings, which embed microscopic musical rules, as well as the composer’s preferences. While many complex systems exhibit emergent properties associated to nonphysical quantities, this work employed physical parameters to trace a connection between the properties of a musical piece as a whole and the psychoacoustic properties of its individual elements.

## Figures and Tables

**Table 1:** Number of combinations of the α ratios that satisfy the degeneracy Equations (18) and (19) as a function of the precision of the α ratios, given in terms of the number of decimal places *d*. Results for 1≤d≤10.

Scale	Up to 24 Semitones		Up to 87 Semitones	
	$f_{j}-f_{i}$	$f_{j}^{2}-f_{i}^{2}$	$f_{j}-f_{i}$	$f_{j}^{2}-f_{i}^{2}$
Just	52 for d≥4	2 for d≥5	208 for d≥4	5 for d≥8
Pythagorean	8 for d≥4	0 for d≥5	47 for d≥5	2 for d≥8
12-TET	0 for d≥5	0 for d≥4	0 for d≥5	0 for d≥8

**Table 2:** Final Shannon entropy of melodic intervals $S_{f}$, maximum Shannon entropy of melodic intervals reached by each melodic line $S_{max}^{*}$, maximum Shannon entropy of melodic intervals generated by the *ambitus* of the corresponding melodic line $S_{max}$, Lagrange multipliers $\lambda_{1}$ and $\lambda_{2}$, mean dissonance level 〈D〉, and mean dissonance level approximated using the Taylor expansion up to second order (Equation (25)) ${\langle D\rangle }^{*}$. Melodic lines marked with “⋆” do not satisfy a linear relation between $\lambda_{1}$ and 〈D〉.

*Melodic Line*	$S_{f}$	$S_{\max}^{*}$	$S_{\max}$	$\lambda_{1}\left(\times 10^{-5}\right)$ $\left[{\mathrm{Hz}}^{-2}\right]$	$\lambda_{2}\left(\times 10^{-7}\right)$ $\left[{\mathrm{Hz}}^{-2}\right]$	〈D〉 $\left(\times 10^{-1}\right)$	${\langle D\rangle }^{*}$ $\left(\times 10^{-1}\right)$
*Violin 1*	7.358	7.378	10.089	0.550	−1.870	1.282	1.278
*Violin 2*	7.213	7.234	10.000	0.570	−0.189	1.215	1.211
*Violin 3*	7.253	7.285	10.000	0.660	−0.895	1.242	1.240
*Viola 1*	6.941	6.953	9.615	1.330	−1.860	1.339	1.333
*Viola 2*	6.935	6.944	9.510	1.500	−1.280	1.381	1.375
*Viola 3*	7.022	7.053	9.716	1.540	−2.200	1.364	1.357
⋆ *Cello 1*	6.888	6.904	9.716	6.300	−18.700	2.795	2.788
⋆ *Cello 2*	6.884	6.899	9.716	6.400	−17.200	2.797	2.790
⋆ *Cello 3*	6.862	6.879	9.716	6.500	−15.100	2.816	2.812
*Violone*	6.779	6.796	9.716	30.000	−34.000	4.900	4.917
⋆ *Harpsichord*	6.779	6.796	9.716	7.400	−4.200	2.596	2.598
*Soprano*	5.055	5.082	8.340	1.940	−2.850	1.470	1.470
*Contralto*	5.247	5.313	8.644	3.250	−6.800	1.591	1.591
*Tenor*	5.443	5.491	7.615	5.100	−6.500	1.893	1.893
*Bass*	5.723	5.787	8.644	7.300	6.450	2.219	2.218
⋆ *Suite 1*	7.069	7.073	10.000	3.500	−5.100	2.528	2.509
⋆ *Suite 2*	7.235	7.248	10.000	3.700	−5.800	2.653	2.631
*Mozart sonata*	6.923	6.935	10.644	0.490	−1.520	1.353	1.357
*First mov. Partita*	7.145	7.145	10.000	0.295	−1.760	1.293	1.294
⋆ *Piccolo concerto*	7.087	7.182	9.288	0.056	0.175	0.749	0.747

## Supplementary Materials

The following are available online at https://www.mdpi.com/1099-4300/21/5/532/s1, Table S1: Frequency ratios used to construct the Pythagorean, the just, and the 12-TET scales, Table S2: Fitting parameters and determination coefficients for the dissonance curves, Table S3: Total number of melodic intervals, *ambitus*, and asymmetry between the number of ascending and descending intervals for each melodic line, Table S4: Determination coefficient $R^{2}$ for the fits of CCDs and histograms to exponential functions, for the quantities $|f_{t+1}-f_{t}|$ and $|f_{t+1}^{2}-f_{t}^{2}|$, Table S5: Fit parameters for the discontinuous asymmetric Laplace distribution function: Real melodic lines, Table S6: Determination coefficient $R^{2}$ for the fit of the bin degeneracy distribution to a power law and to an exponential function, Table S7: Relevant expected values for real melodic lines and the statistical model results, Table S8: Fit parameters for the discontinuous asymmetric Laplace distribution function: Statistical model results, Table S9: Relative error of the fit parameters for the statistical model with respect to those of the real melodic lines, Note S1: Degeneracy equations for the quantities $f_{j}-f_{i}$ and $f_{j}^{2}-f_{i}^{2}$ in the Pythagorean, the just and the 12-TET scales, Note S2: Relation between the uncertainty in the frequency ratios Δα and $\Delta \alpha^{2}$ with the uncertainty in the frequency Δf and the square of the frequency $\Delta f^{2}$, Note S3: Tonal consonance of complex tones: Intervals of equal size played in different locations within the register, Note S4: Relating the average location in the register to an average of dissonance, Note S5: The PD representing a melodic line with the highest diversity of melodic intervals corresponds to the PD of a random melodic line, Note S6: Minimization of the relative entropy subject to constraints, Spreadsheet S1: Data corresponding to the melodic intervals of each melodic line.
